# Tele-Neuro-Rehabilitation in Italy: State of the Art and Future Perspectives

**DOI:** 10.3389/fneur.2020.563375

**Published:** 2020-09-30

**Authors:** Giuseppa Maresca, Maria Grazia Maggio, Rosaria De Luca, Alfredo Manuli, Paolo Tonin, Loris Pignolo, Rocco Salvatore Calabrò

**Affiliations:** ^1^IRCCS Centro Neurolesi Bonino-Pulejo, Messina, Italy; ^2^S. Anna Institute, Research in Advanced Neurorehabilitation, Crotone, Italy

**Keywords:** motor rehabilitation, cognitive training, remote rehabilitation, neurological disorders, neurodegenerative diseases

## Abstract

Current research suggests that the management of neurological diseases, both in adults and children, requires an ever increasing commitment of resources for the national healthcare system (NHS). In Italy, due to the aging of the population, increase in chronicity and morbidity of pathologies, and presence of islands and rural areas, health needs to be supported by innovative technologies. Telemedicine is a method of providing healthcare services at distance, remotely connecting health professionals and patients (or two professionals). In Italy, telemedicine is under development, and the NHS has not yet exploited and independently developed all the possibilities that telemedicine offers. Tele-rehabilitation consists in the use of information and communication technologies for the remote support of rehabilitation services. By allowing “home care,” it represents a valid support during the home rehabilitation process. This review is aimed at evaluating the role of telerehabilitation in Italy, with regard to the motor and cognitive rehabilitation programs applied to neurological pathologies, in both pediatric and adult patients. We screened the studies published between 2010 and 2019 on PubMed, Scopus, Cochrane, and Web of Sciences databases. Using the PICO model, the search combined the terms “telerehabilitation”; “neurological disorders”; “neurodegenerative disease,” “motor telerehabilitation”; “cognitive rehabilitation.” This review showed that telerehabilitation is a promising healthcare tool, as it guarantees continuity of care over time (after discharge) and in space (from hospital to patient's home), especially in patients with stroke. Furthermore, it allows to increase the frequency and intensity of rehabilitation programs, provide individualized rehabilitation treatment in comfortable and familiar environment for patient, monitor and evaluate patients' needs and progress, stimulate patient motivation and achieve better patient satisfaction, verify the results achieved by the patients, and potentially reduce the service costs. Unfortunately, almost all neurorehabilitation studies are characterized by small samples and wide variability of results, and would benefit from standardized procedures, aims and targets. Future telerehabilitation trials should include cost-effectiveness analysis associated with clinical outcomes to better assess the validity of this promising tool.

## Introduction

The National Health System (NHS) undergoes continuous changes, such as reorganization of primary care, integration between different levels of care and continuity of care. Neurological and neurodegenerative disease management, both for adults and children, has a strong impact on the NHS due to the presence of disabilities, with serious economic and social consequences also for families (1, 2). Telemedicine is a method of providing health care services, through the use of Information and Communication Technologies (ICT), in which the health professional and the patient (or two professionals) are far (3). In addition, telemedicine helps overcome patient's mobility problems and reduce NHS costs (4). There are various application areas of telemedicine, including telecardiology, teledermatology, telestroke, and telerehabilitation.

The American Telemedicine Association establishes telerehabilitation as the delivery of rehabilitation services through ICT (5). Telerehabilitation represents an emerging and innovative approach during the home rehabilitation path that the patient undertakes for the improvement of his/her own motor, cognitive or psychological disorders. In particular, tele-neurorehabilitation may provide many types of interventions, including physiotherapy, speech, cognitive and behavioral therapy, occupational therapy, telemonitoring, and teleconsultation (6). Telerehabilitation offers a fair opportunity of access to rehabilitation services for people who live in remote areas or cannot reach the care centers due to physical impairments (7). Indeed, it can guarantee the continuity of care over time (after discharge) and in space (from hospital to patient's home) (8), substantial cost savings (due to the reduction of specialized human resources), an improvement in comfort and patient lifestyle (9), and an increased frequency and adherence to therapy (10). Some authors have observed that patients report high levels of satisfaction with the use of telerehabilitation (7–11), with a decrease in long-term disability, increase in secondary prevention, as well as a better management of the post-acute/chronic phase of disorders (12, 13). Recent studies have shown that the effects of telerehabilitation are comparable to standard care (14–16). A neuroimaging study has demonstrated that telerehabilitation treatments activate the same cortical regions as conventional treatment do (17). However, other studies have highlighted the need to standardize the procedures, aims and targets that characterize this therapeutic modality (18, 19).

Literature data shows that telerehabilitation can be a promising intervention for elderly (20), adults (21), children with neurological diseases (22), as well as for the treatment of motor (23, 24), cognitive (25) and language disorders (26–30). In Italy, the healthcare system could be supported by telemedicine interventions, due to the aging population, the increase in chronicity and morbidity of diseases, and the presence of rural areas, islands, and mountains. However, in Italy, the development of telemedicine is recent, in fact it has been adopted by few hospitals, as disparities exist among the different regions as regards to healthcare provision (2).

Considering the potential of telerehabilitation in the Italian NHS, the aim of this work was to investigate the current use of this innovative tool in Italy, as well as the efficacy of telerehabilitation on motor and cognitive outcomes of neurological disorders, paving the way for the future perspectives of this promising field.

## Methods: Search Strategy

This review sought to investigate the potential of telerehabilitation in the Italian NHS. Studies were identified by searching on PubMed, Web Of Science and Cochrane databases. We defined our combination of search terms using a PICO (population, intervention, comparison, outcome) model. Population was limited to neurological and neurodegenerative patients, including neurodevelopmental disorders; intervention included all telerehabilitation project/protocols realized in the Italian NHS; comparison was evaluated considering the standard cognitive and motor rehabilitation techniques; and outcome included any motor and cognitive improvements shown by the patients, efficacy of treatment or feasibility in terms of cost-benefit or usability of telemedicine system.

**Figure 1:**
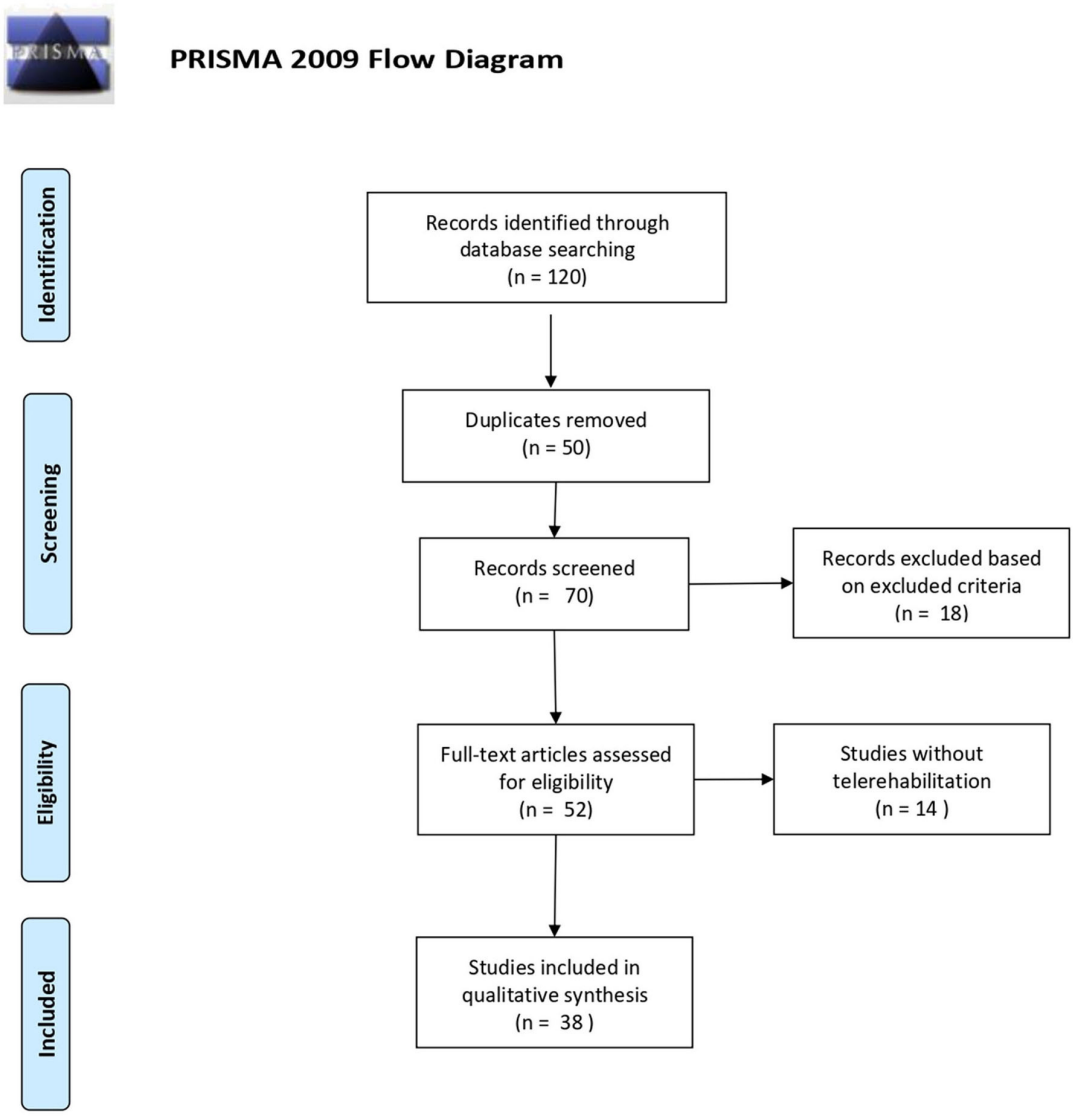
PRISMA 2009 flow diagram (31). For more information, visit www.prisma-statement.org.

The search combined the following terms: “telerehabilitation” OR “remote rehabilitation” AND “neurological disorders” OR “neurodegenerative disorders” AND “motor telerehabilitation” OR “cognitive rehabilitation” AND “Italy” OR “Italian NHS.” All the results of each database between January 2010 and March 2020 were evaluated for possible inclusion (Figure 1). After the removal of the duplicates, all of the articles were evaluated based on the titles and abstracts.

The inclusion criteria were (i) patients with neurological/neurodegenerative disease; (ii) telerehabilitation studied carried out in Italy; (iii) English language; and (v) published in a peer-reviewed journal.

Data extraction was performed on 120 articles, 50 articles were excluded due to duplicates (Figure 1). The data were extracted on the basis of the following data: authors, year, and type of publication (for example, conference proceedings, clinical case), characteristics of the participants involved in the study and purpose of the study.

After an accurate revision of full manuscripts, 38 articles satisfied the inclusion/exclusion criteria and the PICO approach (Table 1).

**Table 1:** The main italian studies reporting either motor or cognitive outcomes following telerehabilitation.

	**References**	**Sample**	**Major findings**
**TELEMOTOR REHABILITATION**			
Postural control and falls	Giordano et al. (24)	145 EG/145 CG elderly patients	The authors performed a trial to evaluate the effects of a home intervention program delivered by a multidisciplinary health-care team on *older people*. The results showed positive effects of the home program.
	Bernocchi et al. (32)	141 EG/142 CG elderly patients	The authors carried out a telerehabilitation program to prevent fall and they observed that their 6-month integrated program was feasible and effective in preventing falls in elderly with chronic diseases and at high risk of falling.
	Gandolfi et al. (33)	38 EG/38CG PD patients	The study evaluate the effects of a remote rehabilitation training on balance, mobility, quality of life, frequency of fall in patients with PD. The authors observed that static and dynamic postural control was improved more in the PD patients receiving VR-based balance training at home.
	Carpinella et al. (34)	21 EG/21 CG PD patients	The study shows the feasibility and efficacy of a new system, the GAMEPAD (GAMing Experience in Parkinson's Disease) for the biofeedback rehabilitation of balance and gait in PD.
	Isernia et al. (35)	30 PD, 32 SM and 45 stroke patients	The authors proposed a motor and cognitive rehabilitation program, the Human Empowerment Aging and Disability (HEAD), and they found that a telehealth approach is both feasible and effective in providing rehabilitation care, ensuring continuity of care and encouraging the autonomy of daily life.
Upper limb	Piron et al. (16)	36 stroke patients	This work found that subjects with post-stroke upper limb deficits exposed to telerehabilitation treatment in a virtual environment could obtain moderately better motor performance than conventional therapy.
	Sgandurra et al. (36)	12 EG/12 CG UCP children	The results of this study showed that telerehabilitation could be promising in upper limb recovery in children with unilateral cerebral palsy (UCP).
	Dallolio et al. (37)	90 EG/90 CG SCI patients	The authors show that in patients with spinal cord injury, motor telerehabilitation would persist over time. Thus, the tool may offer benefits to patients discharged compared to standard treatments in terms of improving functionality.
**TELECOGNITIVE REHABILITATION**			
Pediatric neurological disorders	Corti et al. (38)	32 adolescents adolescents with brain damage	The authors observe that home cognitive telerehabilitation can be very useful in adolescents with congenital or acquired brain damage and with various levels of cognitive functioning.
	Pecini et al. (39)	34 children with Dyslexia	The authors found that rapid automatized naming (RAN) could be a valid tool in children with reading difficulties to empower the cognitive processes underlying reading, implement of intensive, specific, and early interventions, reduce costs for the healthcare system and long waiting lists.
	Simone et al. (40)	16 SM and 40 ADHD children	The study underline the efficacy of a home-based computerized-program for retraining attention. Data suggest that a cognitive rehabilitation program that targets attention is a suitable tool for improving global cognitive functioning in these patients.
Neurological diseases in adulthood	Torrisi et al. (41)	20 EG/20CG post-stroke patients	This work show effectiveness of a virtual reality telerehabilitation system to improve cognitive functioning in 40 post-stroke patients, thanks to a tablet connected remotely with clinicians.
	Maresca et al. (42)	15 EG/15 CG patient with post-stroke aphasia	The authors demonstrate effectiveness of a specific home telerehabilitation program for post-stroke aphasia, using a virtual reality touch-screen tablet.
Neurodegenerative diseases	De Luca et al. (43)	10 EG/10 CG patients with dementia	The study demonstrate that web-based cognitive rehabilitation can be useful in improving cognitive performance, besides psychological well-being, in demented individuals living in home care.
	Realdon et al. (44)	15 EG/15 CG MCI and AD patients	This work showed the effectiveness of a telerehabilitation and telemonitoring home assistance service of MCI and AD patients, to preserve cognitive and motor functioning and increase autonomy in daily life.
	Fabbri et al. (45)	30 EG/30 CG MCI and VCI (vascular cognitive impairment) patients	Results of this study underline on the efficacy of the proposed telerehabilitation to prevent or delay further cognitive decline in MCI/VCI subjects. Indeed, this tool could be promising to counteract cognitive decline and improve both physical functioning and quality of life.
	Dobbs et al. (46)	16 PD patients	The authors created a remotely supervised tDCS protocol for PD patients, combined with cognitive rehabilitation; This protocol allowed the PD patients to receive treatment at home, with reduction of fatigue and improvement of cognitive functioning.
	Alloni et al. (47)	45 neurological patients	The authors propose a tool, CoRe, for cognitive rehabilitation and they underline the importance of support systems for therapists in the provision of remote services, which allow the involvement and participation of users.

## Tele-motor Rehabilitation

Telerehabilitation involves the remote delivery of several rehabilitation services through telecommunication technology, including physiotherapy and occupational therapy, thus, providing assistance to patients forced at home without the physical presence of therapists. Some randomized controlled trials (RCT) on motor function rehabilitation have shown that telerehabilitation can have similar effects to conventional therapy (14, 15). Furthermore, functional magnetic resonance imaging has shown that telerehabilitation activates the same cortical regions as conventional treatment does. Previous telerehabilitation studies for the treatment of the motor function of the upper limb after stroke confirmed these data. Despite these promising results, it is still unclear whether telerehabilitation in the motor field can be effective in neurological patients.

### Postural Control and Falls

Few studies have been carried out in Italy to evaluate falls in a multifactorial way and to develop risk management programs (24, 32). Giordano et al. (24) performed a RCT to evaluate the effects of a home intervention program delivered by a multidisciplinary health-care team on older people, showing the positive effects of this home program. Barnocchi et al. (32) carried out a fall prevention program conducted by a remote physiotherapist on 283 elderly patients. The telerehabilitation program included home physiotherapy training on strength, balance, walking, and a weekly monitoring call by the nurse. The authors observed that their 6-month integrated program was feasible and effective in preventing falls in elderly with chronic diseases and at high risk of falling (32).

Other telerehabilitation studies on postural control have concerned patients with Parkinson's disease (PD) (33). Commonly, this patient population has motor disorders (i.e., up to 75% of people with PD) with postural instability, gait and balance deficits, and an increased risk of falls (48–52). Moreover, dopaminergic drugs have limited effects on postural instability in PD and rehabilitation is the most effective non-pharmacological treatment to reduce the risk of falls (53). However, the growing disability, geographic distances and irregular distribution of rehabilitation services can hinder access to care services for PD patients (54). Given that virtual reality (VR) telerehabilitation has proven feasible and effective in improving various neurological conditions (55, 56), Gandolfi et al. (33) conducted a comparative study assessing the effects of either a standard rehabilitation program or a remote rehabilitation training on balance, mobility, quality of life, frequency of fall in patients with PD. The authors observed that static and dynamic postural control was improved more in the PD patients receiving VR-based balance training at home. On the contrary, the improvements in mobility and balance were greater in those who received conventional rehabilitation. Furthermore, confidence in the performance of outpatient activities, speed of gait, reduction of falls and a better quality of life were positively perceived by all of the patients, independently from the treatment.

Carpinella et al. (34) sought to investigate the feasibility and efficacy of a new system, the GAMEPAD (GAMing Experience in Parkinson's Disease) for the biofeedback rehabilitation of balance and gait in PD. The authors observed that the training was feasible and more effective than traditional physiotherapy on gait performance and the results were maintained for about 1 month. The same positive results were also obtained by Isernia et al. (35), who proposed a motor and cognitive rehabilitation program, the Human Empowerment Aging and Disability (HEAD), on 30 PD patients, 32 individuals with multiple sclerosis (MS) and 45 patients with chronic stroke. The authors found that a telehealth approach is both feasible and effective in providing rehabilitation care, ensuring continuity of care and encouraging the autonomy of daily life (35).

### Upper Limb

In their interesting work, Piron et al. (57) provided five post-stroke patients with a motor telerehabilitation therapy based on increased feedback, suggesting that telerehabilitation could promote learning of the arm's motor skills away from the healthcare facilities, with reduced healthcare costs. In fact, another work by the same authors, carried out on a sample of 36 patients with post-stroke upper limb deficits, found that subjects exposed to telerehabilitation treatment in a virtual environment could obtain moderately better motor performance than conventional therapy (16). These results confirmed the previous pioneeristic study, which was conducted on a smaller group of patients with post-stroke motor deficits, who underwent a motor telerehabilitation program with promising results (6). Taken together, these findings demonstrate that telerehabilitation could be a simple method to treat stroke motor disorders, and could favor hospital discharge.

More recently, other studies have focused on demonstrating the effects of motor tele-rehabilitation in developmental disorders. Sgandurra et al. (36) applied a rehabilitation approach, called UPper Limb Children Action Observation Training, based on action observation training, showing that telerehabilitation can be promising in upper limb recovery in children with unilateral cerebral palsy.

Although several studies have shown that telerehabilitation is effective in improving motor outcomes in different neurological populations, the conclusive evidence on the efficacy of telerehabilitation for the treatment of motor function, regardless of the pathology, has not been reached. However, a positive effect was found for patients following orthopedic surgery, stroke, PD and in the elderly, suggesting that the increased intensity provided by telerehabilitation is a good option to be offered to the patients (23). Furthermore, as observed by Dallolio et al. (37) in patients with spinal cord injury, the results of telerehabilitation would persist over time. Thus, the tool may offer benefits to patients after discharge, as compared to standard treatments, in terms of improving functionality, although more studies are needed to advance the neurological field.

## Tele-Cognitive-Rehabilitation

Neuropsychological rehabilitation stimulates cognitive functions to achieve the patient's maximum degree of autonomy and independence. Cognitive rehabilitation therapy has been defined as systematic and functionally oriented therapeutic cognitive activities directed to achieve functional changes by reestablishing or strengthening previously learned patterns of behavior or establishing new patterns of cognitive activity or compensatory mechanisms for impaired neurological systems. Thus, similar to the other types of rehabilitation therapy, cognitive rehabilitation includes both restorative and compensatory approaches. Cognitive rehab may be provided in a traditional way (i.e., the paper and pencil approach) or using innovation technology. There are various cognitive rehabilitation software, with different characteristics, such as the ability to adapt the level of difficulty of the exercises to the patient's performance and to choose sets of exercises based on the patient's deficit (58, 59). In the last decades, advances in ICT have led to the development of platforms and applications to enable patients' cognitive rehabilitation therapy at home. A crucial role is played by the professional and his/her abilities to involve the patients in their treatment process.

Rosso et al. (60) designed a platform based on the principles of cognitive rehabilitation and operators' decision-making. The latter has proven very important, as it affects the treatment by personalizing the exercises, the settings of cognitive exercises, and feedback.

Cognitive telerehabilitation has been used in the treatment of different neurological conditions and to improve different cognitive domains.

### Pediatric Neurological Disorders

Technology-based treatments represent a promising field for multidisciplinary rehabilitation of children with acquired brain injury (ABI). Corti et al. (22) underlined in a recent review that telerehabilitation is promising to enhance cognitive and behavioral domains remotely. Traditional cognitive rehabilitation is known to have some limitations related to the time, costs, and accessibility of patients to these services (61–63). Therefore, the use of rehabilitation technology could also allow the provision of services remotely and in a non-medical environment (61, 64).

Zampolini et al. (61) have found that telemedicine allows for continuity of care in the treatment of neurodevelopmental disorders, limiting the time and economic needs of families and institutions. It also allows for precise monitoring of patient performance through online monitoring. Indeed, in another study, Corti et al. (38) investigated the feasibility of home computerized cognitive training in a group of 32 subjects aged between 11 and 16 years, observing that home cognitive training can be very useful in adolescents with congenital or acquired brain damage and with various levels of cognitive functioning.

Another field of application of cognitive telerehabilitation in developmental age is dyslexia. Pecini et al. (39) compared a training (Reading Trainer) working on the reading impairment with one [Run the rapid automatized naming (RAN)] working on the RAN impairment. The authors found that RAN could be a valid tool in children with reading difficulties by passing the use of alphanumeric stimuli, but empowering the cognitive processes underlying reading. This aspect could be useful for the implementation of intensive, specific, and early interventions, which in the traditional approach entail a series of complications, such as high costs for the healthcare system, long waiting lists that often lead to delayed treatments (65–67). In another study by the same authors on transparent spellings, it was found that software for a home-rehabilitation of reading disorders in children with dyslexia can involve the different linguistic, visual, and attentional processes, and integrate the components into a complex activity, such as reading (68). According to these studies, various authors (65, 68–71) have observed that telerehabilitation can be a promising approach potentially affecting multiple cognitive and linguistic components at the basis of normal and compromised reading, given that dyslexia has a “multifunctional deficit model.” Thus, it is believed that ICT can effectively speed up reading aloud after only a few months, and this can facilitate the automation of reading processes (65, 68).

In fact, the home telerehabilitation software allows intensive and daily interventions, and the levels of difficulties and settings are managed by the operator to improve the automation process (72, 73), inserting activities with the difficulty of the exercise above the performance of the child, making the task not only stimulating but also easy to guarantee success (74). In addition, the adaptation of the intervention also allows continuous feedback to the child and stimulates greater self-control of competence (75–77).

Finally, recent applications of telerehabilitation in pediatric age have been aimed at the treatment of cognitive deficits in pediatric-onset multiple sclerosis (POMS) and in children with attention deficit hyperactivity disorder (ADHD). In fact, Simone et al. (40) performed a pilot study on these pediatric populations to evaluate the effectiveness of a home computerized attention program. The authors found that the tool was useful for improving overall cognitive functioning in patients with POMS, but it had a minor effect in patients with ADHD.

### Neurological Diseases in Adulthood

Stroke is a leading cause of mortality in industrialized countries (78), as well as the leading cause of long-term disability in adults. The prevalence of post-stroke cognitive dysfunction ranges from 23 to 55% within 3 months of the stroke onset, and decreases between 11 and 31% after 1 year (79–81). The main post-stroke cognitive deficits involve attention and concentration (82), memory (83), spatial awareness (84), perception (85), praxis (86), and executive functioning (87), with a significant reduction in autonomy of daily life and quality of life. In recent years, VR tele-rehabilitation has been used to manage this patient population, with the aim of reducing healthcare costs and encouraging continuity of care. A recent study (41) has evaluated the effectiveness of a VR telerehabilitation system to improve cognitive functioning in 40 post-stroke patients. After an initial training phase, the patients continued rehabilitation at home thanks to a tablet, connected remotely with clinicians. Data showed the effectiveness of telerehabilitation for the treatment of cognitive disorders following stroke, with an improvement in global cognitive functioning, attentional processes, verbal fluency, short-term memory and mood. These results seem promising for the management of cognitive disorders in stroke patients, through continuity of care that allows maintenance of the recovery achieved, as well as to reduce hospitalizations and improve the patient's emotions and well-being (41).

Almost 30% of patients with ischemic and hemorrhagic stroke have an aphasic syndrome in the acute phase (88–90), and when severe, aphasia may persist all lifelong. Aphasia has a significantly negative impact on well-being, independence, social participation, quality of life, and it is often associated with severe depression (91, 92). Early therapy is important for promoting language recovery, which is essential for daily communication and social participation (26). Maresca et al. (42) assessed the effectiveness of a specific home telerehabilitation program for post-stroke aphasia, using a VR rehabilitation system touch-screen tablet in 30 patients. The study showed that telerehabilitation could be one solution for the treatment of aphasic patients after discharge, by promoting the continuity of care, monitoring results, and improving linguistic abilities, mood and psychological well-being.

Another area of the application of telerehabilitation in the stroke field concerns visual impairments, with regard to hemianopia, which may affect cognitive functioning and neurological recovery (93). In fact, the presence of visual defects can be very debilitating and interfere with daily life, negatively affecting the prognosis of stroke and the independence of the person, with a significant negative emotional impact and social implications. Tinelli et al. (94) developed an audiovisual telerehabilitation system, based on the brain's multisensory ability, in order to provide a new tool to improve eye movements toward the blind half-field. This tool has been tested on three adult patients, leading to improvements in visual detection skills with long-term effects. Therefore, the results showed that in stroke, telerehabilitation could be a promising, convenient and accessible tool for more patients, relieving the NHS (94). In addition, remote rehabilitation allows intense and prolonged intervention for patients who may not be able to access the healthcare system.

The potential of telerehabilitation could be confirmed by Calabrò et al. (95), who are conducting a multicenter study on the role of cognitive telerehabilitation in individuals with ABI, evaluating the functional recovery of patients, psychological well-being, the burden of caregivers with a cost-effectiveness analysis.

### Neurodegenerative Diseases

Another neurological field of increasing interest for telerehabilitation concerns neurodegenerative diseases, also considering the limited efficacy of drugs in these pathologies. According to the World Alzheimer Report (96), dementia affects 46.8 million people worldwide, with an increasing burden on society and the families. The lack of drugs that can stop or slow the course of the disease have encouraged non-pharmacological approaches for people with/at risk of dementia, such as in Alzheimer Diseases (AD), Mild Cognitive Impairment (MCI), Frontotemporal Dementia (FTD), and PD. De Luca et al. (43) assessed the effectiveness of cognitive training on 20 people with dementia living in a nursing home, observing that web-based cognitive rehabilitation can be useful for improving cognitive performance, as well as psychological well-being. Similar results were obtained by Cotelli et al. (97), who showed that cognitive telerehabilitation interventions have the same efficacy as face-to-face rehabilitation in patients with MCI, AD and FTD. Moreover, Realdon et al. (44) showed the efficacy of a telerehabilitation and telemonitoring home assistance service to preserve cognitive and motor functioning and increase autonomy in daily life in 30 MCI and AD patients. New research is taking place to evaluate the effectiveness of telerehabilitation in preventing or delaying further cognitive decline and improving physical functioning and quality of life (44, 45).

As for PD, recent studies have observed that telerehabilitation allows the delivery of combined treatments involving cognitive stimulation and transcranial direct current stimulation (tDCS). The latter is a non-invasive brain stimulation technique that has been shown to improve several symptoms of neurological disorders, such as depressed mood, fatigue, motor deficits and cognitive dysfunction. Dobbs et al. (46) created a remotely supervised tDCS (RS-tDCS) protocol for PD patients to increase accessibility of this tool, reducing the burden on physician, patient and caregiver. RS-tDCS combined with cognitive rehabilitation was feasible, and it allowed the PD patients to receive treatment at home, with consequent reduction of fatigue and improvement of cognitive functioning. Hence, according to the authors (46), RS-tDCS after-effects can be generalized to provide tDCS for home rehabilitation to patients with other neurological disorders, including multiple sclerosis (MS).

MS is a chronic immune-mediated disease of the central nervous system, and it is a major cause of disability in young adults. The disease can lead to motor, sensory, cognitive and behavioral anomalies, and recently there has been a growing interest in the development of innovative rehabilitation treatments, even outside the hospital environment. Di Tella et al. (98), in their systematic review and meta-analysis, evaluated the effectiveness of integrated telerehabilitation on motor, cognitive and participation outcomes in people with MS. The authors reported that MS patients may benefit from motor telerehabilitation, but have low cognitive and participation outcomes. Further research is needed to confirm the results and develop integrated systems that allow addressing the user's multiple requests. For this reason, Alloni et al. (47) proposed a tool, i.e., the CoRe, for cognitive rehabilitation of a variety of diseases, customizing the exercises according to the preferences and performance of the patients. The authors underlined the importance of specific support systems for therapists in the provision of remote services, which allow the involvement and participation of the users (47).

## Telemonitoring, Teleassistance, and Beyond

Home telemonitoring of chronic diseases is a promising approach that provides accurate and reliable patient's data, influencing attitudes and behaviors toward daily life, potentially improving his/her medical conditions (99). In recent years, various studies have encouraged the use of remote monitoring for chronic diseases at home. Neuromuscular diseases (NMD) present with progressive muscle deficits, with consequent loss of walking, difficulty swallowing and weakness of the respiratory muscles, and such disorders deserve a continuous follow up.

Portaro et al. (100) carried out a study to evaluate the effectiveness of telemedicine in monitoring 4 patients with facio-scapulo-humeral dystrophy (FSHD), a hereditary disease characterized by variable and asymmetrical involvement of the muscles of the face, trunk, upper and lower limbs. The patients had a severe form of FSHD (with chronic respiratory failure) and lived on a small island (Lipari, Sicily), far away from the treatment center. The authors pointed out that the telemonitoring system was simple to use, efficient and effective for home management of these patients with FSHD, and allowed reduction of hospitalizations.

Another interesting study was conducted by Trucco et al. (101), for the home management through telemedicine of adult patients with a ventilator-dependent NMD. The 2-year longitudinal multicenter study found that telemedicine was effective in improving home management of respiratory exacerbations in the young patients, and it was overall well-tolerated.

Also, telemonitoring has been found effective in other chronic neurological conditions. Marzianiak et al. (102) observed the effects of telemonitoring *via* an e-Health app in MS patients. The authors pointed out that this app can improve predicted outcomes, access to treatment as well as disease information. In particular, it has been found that the e-Health app has allowed an active participation in all aspects of the self-management of the pathology, including the control of adherence to treatment, changes in bladder and bowel habits, activities of daily live and mood. According to the authors, telemonitoring devices may be important in the management of chronic diseases, such as MS, to simplify the multidisciplinary approach (102). These tools could facilitate remote monitoring of symptoms, adverse events and patient outcomes, and might allow efficient use of resources and limit the time spent in hospital, permitting a more timely intervention of scheduled face-to-face visits. Moreover, telemedicine can also be a useful approach for assistance of elderly with chronic diseases. Maresca et al. (20) treated 22 elderly patients with a multidisciplinary telemedicine approach to improve their quality of life. The authors observed that tele-assistance could be an effective care tool for the elderly, promoting remission of depressive symptoms and improving social functioning, cognitive levels and eating habits, helping in preventing the exacerbations of pre-existing chronic diseases. In fact, another work by the same authors (103) suggested that telemedicine (telemonitoring/teleassistance) could be useful to improve health and quality of life of disadvantaged elderly people, suffering from severe comorbidity or living far from health services. Moreover, patients reported high levels of satisfaction with the service, although the system was not easy to use (103).

In a systematic review performed by Nordio et al. (104), it has been observed that tele-rehabilitation improves the patient's adherence to the treatment. High levels of satisfaction and adherence to telemedicine treatment were found also by Piron et al. (11) in post-stroke patients. These works reinforce the hypothesis that remote services are a feasible alternative to standard care (11, 18). Other authors have highlighted the advantages of telemedicine, especially of tele-rehabilitation services, such as reducing costs (both for public health and for patients) and increasing the access to care for patients living in isolated areas, where traditional rehabilitation services may not be easily accessible (24, 69).

However, Perretti et al. (105) found that the patients were skeptical about the efficacy of a therapy mediated by a telemedical system (pc, videoconferencing) without a direct and vis-a-vis interaction with the healthcare professionals. For this reason, the authors suggest both a monitoring patient feedback (to adapt rehabilitation techniques and approaches to his/her needs), and the need for adequate training of the users involved. In line with these authors, Calvaresi et al. (106) made a focus group with physiotherapy and telerehabilitation experts, identifying as the strength of an appropriate approach the possibility of (i) contextualizing the scenario, (ii) promptly solving planning and problem-solving uncertainties, and (iii) offering reliable sources of information and controls. In addition to these proven positive characteristics, limitations were found, such as incompatibility with real-time operating systems, and inability to perform interventions in integrated devices (106). For this reason, new and larger studies are needed to demonstrate the positive effects and evaluate the strengths and limitations of telemedicine in the rehab field.

## Ongoing Projects and Future Perspectives

**Figure 2:**
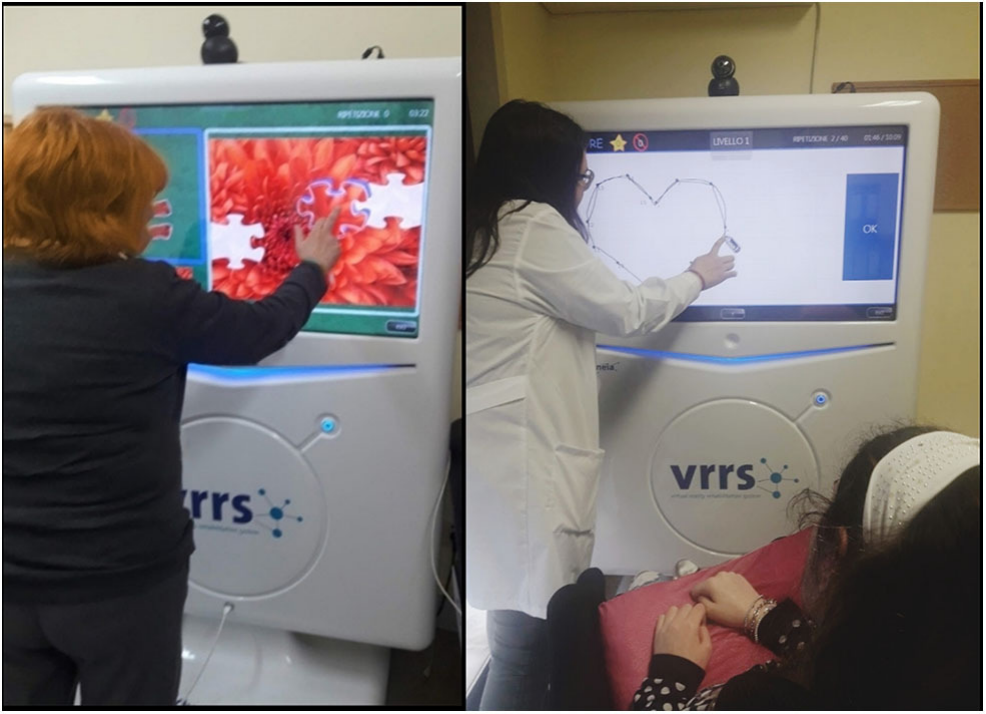
The VRRS system: the Italian Telerehab tool used for both motor and cognitive rehabilitation.

Currently, in Italy, some health care centers use telerehabilitation for provision of health services. In particular, Bramanti et al. illustrated an ICT model implemented in Sicily to ensure the continuity of care, which is very difficult because of the geographical characteristics and socio-economic problems of the island. In fact, the IRCCS Centro Neurolesi “Bonino-Pulejo” of Messina, together with the Sicilian Government and the Ministry of Health, has incorporated the telehealth system into the long-term post-stroke patient care program to provide continuous rehabilitation and reduce disability (8). The project follows a hub-and-spoke system, consisting of a main supplier center (hub) integrated by secondary institutions (spokes), that offer more limited services. Patients are followed during their individual rehabilitation path (from intensive rehabilitation to home rehabilitation). Through a system of telerehabilitation with virtual reality (i.e., the Virtual Reality Rehabilitation System—VRRS, Khymeia, Italy, shown in Figure 2), patients are treated with specific programmed exercises, which are continuously modified according to the individual cognitive and motor state. VRRS is a virtual reality system that allows telerehabilitation. VRRS is used in two ways (i) online, with a live interaction between the patient and the therapist *via* videoconference and (ii) offline, in which the patient performs the exercises remotely with the help of a virtual assistant, while the therapist can view results later (8).

Following this positive experience, the Italian Ministry of Health founded in 2015 a National Telerehabilitation network involving the main Research Institutes (i.e., the “Istituti di Ricovero e Cura a Carattere Scientifico,” IRCCS) and aimed at assessing the efficacy and cost-effectives analysis of telerehab in the treatment of different neurological disorders, including stroke, dementia, PD, and MS. Also, Calabrò et al. (107) underlined the potential of telemedicine in guaranteeing distance training and comparison between the healthcare professionals, encouraging the relationship between the Hub center and the Spokes. In fact, these centers are constantly connected thanks to telemedicine: the different specialists support each other, both for diagnosis and for rehabilitation. Furthermore, this program allows the application of standard protocols with fairer and uniform health services (107).

## Authors' Point of View and Conclusions

We could hypothesize that telemedicine services, when used correctly, can contribute to a transformation of the healthcare sector and business models. Telemedicine can assist in an early and protected hospital discharge, which is nowadays necessary because of the increase in life expectancy, and the increase in chronic and post-acute illnesses. Moreover, telerehabilitation can be useful to avoid a direct contact between clinicians and patients, and this issue is of outmost importance during pandemics, like COVID-19 is (108).

For this reason, information technologies applied to medical science can be useful in countering today's socio-health problems. The analysis of Italian telemedicine studies and projects shows that they have led to satisfactory results and have provided solutions to hitherto unresolved problems. Unfortunately, the samples of these studies are small, thus making the results not very relevant for a substantial economic analysis. Specifically, studies on telerehabilitation suggest that it is not yet widely disseminated, despite scientific results are increasingly suggesting its effectiveness. One of the reasons may be the difficulty of creating software suitable for a remote therapeutic setting. In fact, flexible devices that are adaptable to different types of deficits and ample connectivity are needed to better reach users at home. For this reason, the effectiveness of telerehabilitation services compared to standard care is still under debate. All the research on telerehabilitation considered in this study highlights the need to standardize the procedures, purposes and targets that characterize this therapeutic modality. However, considering the growing burden of care and the need to provide adequate and continuous services to chronic patients, telerehabilitation is becoming an interesting and promising model of care. To understand if the infrastructures can be adequate for carrying out such rehabilitation services, valid tests must be designed and carried out to avoid wasting resources and inconclusive results.

Future telerehabilitation studies should include cost responsibility and cost-effectiveness analysis associated with clinical outcomes.

In conclusion, according to the Italian experience with telemedicine applied to the neurorehab field, the studies of this review support the development of telerehabilitation, but the quality of research needs to be significantly improved to clarify benefits and risks of remote assistance. Studies, protocols and guidelines about teleneurorehabilitation are needed from all over the world in order to share the different experiences and better assess the cost-effectiveness analysis of this promising tool, potentially improving healthcare services, especially during pandemics.

## Data Availability Statement

The raw data supporting the conclusions of this article will be made available by the authors, without undue reservation.

## Ethics Statement

Written informed consent was obtained from the individuals for the publication of any potentially identifiable images or data included in this article.

## Author Contributions

RC, GM, MM, and PT: substantial contributions to the conception and design of the work, interpretation of data, revising the work critically for important intellectual content, final approval of the version to be published, and agreement to be accountable for all aspects of the work in ensuring that questions related to the accuracy or integrity of any part of the work are appropriately investigated and resolved. RD, AM, and LP: acquisition and analysis of data, drafting the work, final approval of the version to be published, and agreement to be accountable for all aspects of the work in ensuring that questions related to the accuracy or integrity of any part of the work are appropriately investigated and resolved. All authors contributed to the article and approved the submitted version.

## Conflict of Interest

The authors declare that the research was conducted in the absence of any commercial or financial relationships that could be construed as a potential conflict of interest.
